# Fenugreek Counters the Effects of High Fat Diet on Gut Microbiota in Mice: Links to Metabolic Benefit

**DOI:** 10.1038/s41598-020-58005-7

**Published:** 2020-01-27

**Authors:** Annadora J. Bruce-Keller, Allison J. Richard, Sun-Ok Fernandez-Kim, David M. Ribnicky, J. Michael Salbaum, Susan Newman, Richard Carmouche, Jacqueline M. Stephens

**Affiliations:** 1 Pennington Biomedical Research Center, Louisiana State University System, Baton Rouge, LA 70808 USA; 2 Department of Plant Biology, Rutgers University, New Brunswick, NJ 08901 USA

**Keywords:** Dyslipidaemias, Obesity

## Abstract

Fenugreek (*Trigonella foenum-graecum*) is an annual herbaceous plant and a staple of traditional health remedies for metabolic conditions including high cholesterol and diabetes. While the mechanisms of the beneficial actions of fenugreek remain unknown, a role for intestinal microbiota in metabolic homeostasis is likely. To determine if fenugreek utilizes intestinal bacteria to offset the adverse effects of high fat diets, C57BL/6J mice were fed control/low fat (CD) or high fat (HFD) diets each supplemented with or without 2% (w/w) fenugreek for 16 weeks. The effects of fenugreek and HFD on gut microbiota were comprehensively mapped and then statistically assessed in relation to effects on metrics of body weight, hyperlipidemia, and glucose tolerance. 16S metagenomic analyses revealed robust and significant effects of fenugreek on gut microbiota, with alterations in both alpha and beta diversity as well as taxonomic redistribution under both CD and HFD conditions. As previously reported, fenugreek attenuated HFD-induced hyperlipidemia and stabilized glucose tolerance without affecting body weight. Finally, fenugreek specifically reversed the dysbiotic effects of HFD on numerous taxa in a manner tightly correlated with overall metabolic function. Collectively, these data reinforce the essential link between gut microbiota and metabolic syndrome and suggest that the preservation of healthy populations of gut microbiota participates in the beneficial properties of fenugreek in the context of modern Western-style diets.

## Introduction

Obesity linked to Western-style diets is the prototypical ailment of the modern era. Obesity currently affects more than 35% of Americans^1^; and in addition to ties with type 2 diabetes and cardiovascular disease, obesity increases the risk of all-cause mortality and exacerbates anxiety and depression^2–5^. While search for effective obesity treatments has become a priority in biomedical research, available pharmacological options for obesity are undermined by issues related to toxicity and off-target side^6^. Herbal medicine or phytotherapy has long been a source of traditional medicinal remedies, and indeed, interest in generally regarded as safe (GRAS) plant materials for the clinical treatment of obesity is growing (reviewed in^7,8^). Fenugreek (*Trigonella foenum-graecum*) is an annual herbaceous plant and a staple of traditional health remedies to treat hyperlipidemia and diabetes^9–12^, as well as mood disorders^13^. Laboratory studies demonstrate protective effects of fenugreek on diabetes^14–18^, and suggest that potential mechanisms might include inhibition of intestinal glucose absorption^14–16^, delayed gastric emptying^15^, and/or insulinotropic activity^19,20,17,18^. Protective effects of fenugreek on cholesterol and hyperlipidemia^21^ might be based on modulation of hepatic steatosis^22–26^, inflammation^26–28^, and/or oxidative stress secondary to diabetes^29–32^. While the exact mechanisms whereby fenugreek or its constituents confers metabolic resiliency are unknown, data show that fenugreek administration can also modulate intestinal microbiota, which can in turn impact metabolic physiology^33,34^.

A remarkably mutualistic relationship exists between gut microbiota and their mammalian hosts, with microbiota providing protection against ingested pathogens, neutralizing carcinogens, and metabolizing otherwise inaccessible lipids and polysaccharides into potent bioactive metabolites^35^. Sequencing data show that modern high fat/calorie diets can disrupt gut microbiota, reducing bacterial diversity and upsetting the balance of pathogenic and commensal bacteria^36^. Data from our lab and others show that such diet-induced gut dysbiosis is sufficient to impair both metabolic and neurologic function^37,38^, suggesting that preservation of healthy gut microbiota could offset the pathophysiologic effects of high fat diets^39^. As fenugreek has indeed been shown to modulate intestinal bacteria in several models^33,34,40^, studies were designed to determine if fenugreek could offset the effects of a high fat diet on gut dysbiosis, and to establish the relationship of fenugreek-shaped gut microbiota to clinically relevant metrics of metabolic function. To this end, data from our previously published study on the effects of fenugreek on mice given a high fat diet were extended to include sequencing and statistical assessment of gut microbiota. As reported in our previous study, high fat or nutritionally matched low fat diets supplemented with ground fenugreek seeds (2% w/w) were administered to male C57BL/6J mice for 16 weeks, and the metabolic effects of the various diets on adiposity, glycemic control, and hyperlipidemia were quantified^41^. Metagenomic sequencing of fecal microbiota collected from mice was conducted, and diet-related changes in gut microbiota were statistically analyzed in relation to established metrics of metabolic function.

## Results

### Fenugreek improves glucose tolerance and dyslipidemia in mice given high fat diet

Data in this manuscript is built on initial publication of the effects of whole fenugreek seed supplementation (2% w/w) on overall metabolic function in the context of a 16-week trial of high fat diet consumption^41^, and thus previously published data are only summarized in this report. Briefly, data show that fenugreek supplementation increased HDL and decreased LDL cholesterol levels in high fat fed-mice (Table 1). Furthermore, fenugreek significantly improved glucose tolerance (as measured 40 minutes after oral glucose loading), but did not affect HFD-induced changes in total cholesterol, body weight, amount of body fat, or fasting blood glucose (Table 1). Fenugreek administration did not cause changes in food intake^41^.

**Table 1:** Summary of fenugreek-induced metabolic resiliency: decreased hyperlipidemia and improved glucose tolerance.

	CD	CD/FG	HFD	HFD/FG
Total Cholesterol (mg/dl)	148.9 ± 45.7	136.2 ± 31.7	255.4 ± 32.9***	245.7 ± 26.3
LDL Cholesterol (mg/dl)	9.62 ± 2.6	8.48 ± 2.1	17.98 ± 4.7***	13.83 ± 4.3^#^
HDL Cholesterol (%TC)	45.15 ± 12.7	44.86 ± 5.8	28.28 ± 3.3***	33.3 ± 5.1^#^
Body Weight (gr)	31.78 ± 3.2	31.57 ± 2.6	48.73 ± 2.7***	50.03 ± 2.2
Body Fat (gr)	5.15 ± 1.8	5.04 ± 1.2	16.61 ± 1.3***	16.86 ± 1.3
Fasting Blood Glucose (mg/dl)	153.4 ± 16.8	149.4 ± 23.4	211.1 ± 16.8***	219.1 ± 21.1
Glucose Tolerance (40 min)	234.0 ± 49.3	213.5 ± 23.3	386.1 ± 89.9***	311.6 ± 75.8^#^

Adult male C57Bl/6 mice were given high fat (HFD) or nutritionally matched control diet (CD) with or without fenugreek (FG; 2% w/w), and subject to measures of metabolic function as described in Methods. Statistically significant differences in metabolic parameters in HFD-fed mice as compared to CD-fed mice are mice are noted by ***(p < 0.001), while significant changes in mice given HFD/FG as compared to HFD-fed mice are noted by ^#^(p < 0.05). Adapted from previously published data^41^.

### Fenugreek and high fat diet exert pronounced effects on gut microbial composition

**Figure 1:**
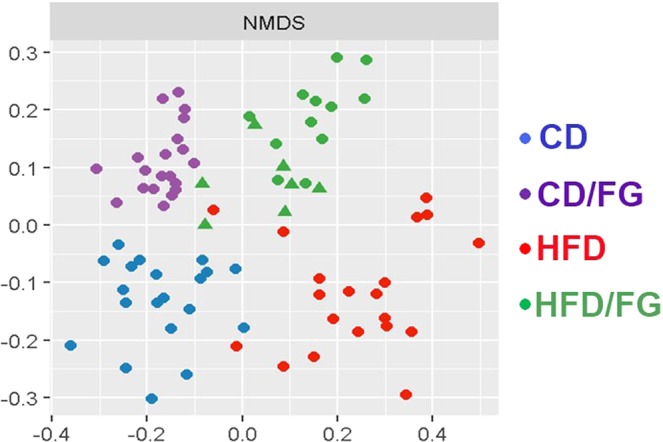
Fenugreek changes intestinal microbial populations in mice. Fecal microbiome populations from CD, CD/FG, HFD, and HFD/FG mice were analyzed using 16S rRNA sequencing, and multi-dimensional scaled principal coordinate analysis were used to visualize UniFrac distances of fecal samples from individual recipient mice. Samples from CD, CD/FG, HFD, and HFD/FG mice are depicted as blue, purple, red, and green symbols, respectively.

**Figure 2:**
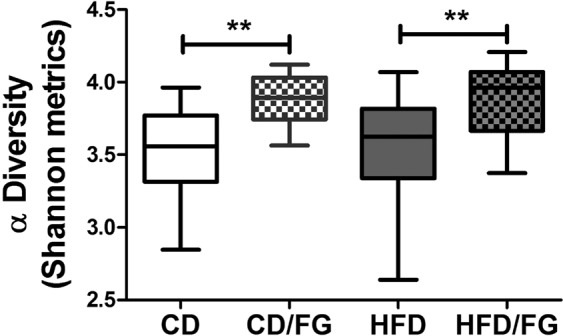
Fenugreek increases overall intestinal microbial diversity. Fecal microbiome populations from CD, CD/FG, HFD, and HFD/FG mice were analyzed using 16S rRNA sequencing, and box plots were generated to depict differences in Shannon α-diversity. Data show that mice supplemented with 2% fenugreek in their feed exhibited a statistically significant (**p < 0.01) increase in α-diversity compared to mice given CD or HFD alone.

The impact of fenugreek on intestinal microbiota was determined by 16S sequencing of fecal samples isolated from mice at euthanasia as described in Methods. Initial weighted and unweighted Unifrac phylogenetic analyses reveal that the microbiomes of fenugreek-fed mice were significantly different from non-fenugreek mice under both control diet and high fat-fed conditions (Table 2). Indeed, the magnitude of the effects of fenugreek were similar in that of the high fat dies as compared to control diet (Table 2). These robust shifts in beta-diversity were also apparent on principal component analysis plots generated from normalized read count data (Fig. 1). Finally, data show that fenugreek also significantly increased alpha diversity (Shannon metrics) in both control diet and high fat-fed mice (Fig. 2).

**Table 2:** Differences in microbiota community composition in mice with CD- and HFD-shaped microbiota with and without fenugreek.

	Comparison	Score	P value
Weighted Unifrac	CD vs CD/FG	0.65012	<0.001***
	HFD vs HFD/FG	0.61915	<0.001***
	CD vs HFD	0.796987	<0.001***
Unweighted Unifrac	CD vs CD/FG	0.90777	<0.001***
	HFD vs HFD/FG	0.885073	0.002009**
	CD vs HFD	0.952579	0.001009**

Operational taxonomical units (OTU) were identified based on sequence clustering as described in Methods, and generation of a read count table was performed with the software package ‘usearch’. Statistical tests for differential representation were performed with tools incorporated in ‘mothur’, and statistically significant differences in microbiota community composition between groups were detected using both weighted and unweighted Unifrac phylogenetic analysis tools.

### Fenugreek can correct the dysbiotic effects of high fat diet on intestinal microbial populations

**Figure 3:**
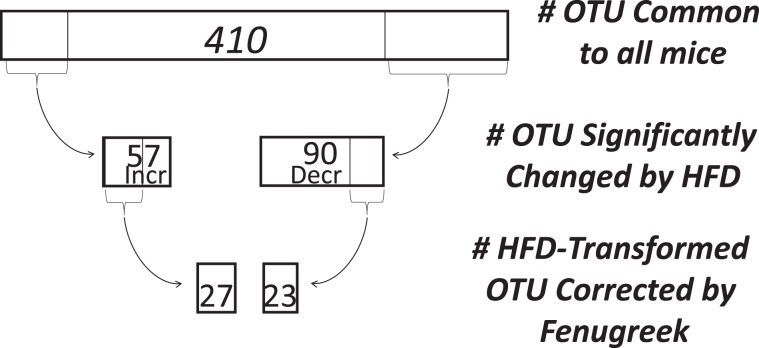
Graphical representation of fenugreek’s ability to reverse HFD-induced changes in individual intestinal microbiota. The ability of high fat diet to significantly alter the representation of individual taxa was assessed as described in Methods showing that out of 410 Core OTUs, 57 were significantly increased and 90 significantly decreased by HFD. Fenugreek supplementation significantly reversed the effects of HFD on 50 of these OTUs by reducing the representation of 27 HFD-increased OTUs and bolstering the representation of 23 OTUs reduced by HFD.

To assess the impact of fenugreek and HFD on gut microbial composition in greater statistical detail, a differential analysis of count data was conducted using DESeq. 2. Specifically, the specific individual operational taxonomic units (OTUs) whose relative representation was significantly (p < 0.05 adjusted with Benjamini-Hochberg correction) changed by high fat diet were identified using DESeq. 2. These analyses revealed that out of 410 Core OTUs (identified in all mice), the relative representation of 147 individual OTUs was significantly different in HFD-fed mice as compared to CD-fed mice (Fig. 3); with 57 increased and 90 decreased, respectively, by HFD. A similar DESeq. 2 analysis of these 147 “HFD-transformed” taxa was conducted to identify those that were significantly affected by fenugreek such that the direction of the change induced by high fat diet was reversed. This ivvestigation of “fenugreek-corrected” taxa revealed that fenugreek corrected the effects of HFD on 50 of these OTUs by reducing the representation of 27 HFD-increased OTUs and augmenting the representation of 23 OTUs reduced by HFD (Fig. 3). These analyses reflect the robust effect of both fenugreek and HFD on gut microbiota, and show that fenugreek is able to significantly correct much (greater than 34%) of the dysbiotic effects of HFD.

### Representation of Fenugreek-corrected taxa largely predicts overall metabolic function

In the final set of analyses, the 50 fenugreek-corrected taxa whose representation was skewed in one direction by HFD but in the opposite direction by fenugreek was examined in relation to metabolic function. Specifically, to determine if the relative representation of fenugreek-corrected taxa could be used to predict metabolic resiliency, the statistical relationship of fenugreek-corrected taxa representation to the metrics of metabolic function listed in Table 1 was determined. To this end, a matrix was built containing OTU count data for all 50 fenugreek-corrected taxa along with all metabolic data depicted in Table 1, including those indices not affected by fenugreek. Pairwise Pearson correlations indicate that the relative expression of many of these 50 OTUs were highly predictive of metabolic function. For example, of the 23 taxa decreased by HFD but increased with fenugreek supplementation, 8 taxa (all in the fermicutes phylum) significantly correlated with at least 1 metric of metabolic function (Table 3, see Supplementary Table 1 for additional details (log2FC and Pearson r values) on HFD-decreased, fenugreek corrected taxa). Likewise, of the 27 taxa increased by HFD but decreased with fenugreek supplementation, 20 taxa significantly correlated with selected metrics of metabolic function (Table 4, see Supplementary Table 2 for additional details (log2FC and Pearson r values) on HFD-increased, fenugreek corrected taxa). It is important to note that the representation of fenugreek-corrected taxa correlated frequently with aspects of metabolic function (e.g., body weight, body fat, total cholesterol, fasting blood glucose) that were not significantly improved in fenugreek-treated mice.

**Table 3:** OTUs that predicts metabolic decline in high fat-fed mice.

Correlation (Pearson) of HFD-Decreased, Fenugreek-Corrected OTU’s to Metrics of Metabolic Function							
Individual OTUs Decreased by High Fat Diet (HF) Corrected by FG	Total Chol.	LDL Chol.	HDLChol.	Body Weight	Body Fat	Fasting Glucose	Glucose Tolerance
Firmicutes/Clostridia/Clostridiales/Lachnospiraceae/Clostridium_XlVa/Species1	*ns*	*ns*	*ns*	*ns*	0.0014	*ns*	0.0001
Firmicutes/Clostridia/Clostridiales/Lachnospiraceae/Clostridium_XlVa/Species2	*ns*	*ns*	*ns*	0.0027	0.0025	*ns*	*ns*
Firmicutes/Clostridia/Clostridiales/Lachnospiraceae/Clostridium_XlVa/Species3	0.0001	0.0034	0.0011	*ns*	*ns*	0.0006	0.0001
Firmicutes/Clostridia/Clostridiales/Ruminococcaceae/Flavonifractor	1.2E-05	0.0005	0.0001	1.6E-06	2.1E-06	2.2E-05	1.9E-05
Firmicutes/Erysipelotrichia/Erysipelotrichales/Erysipelotrichaceae/Turicibacter	0.0004	*ns*	0.0024	*ns*	*ns*	*ns*	0.0015
Firmicutes/Clostridia/Clostridiales/Ruminococcaceae/Oscillibacter	0.0002	*ns*	1.3E-05	*ns*	4.0E-05	0.0002	4.2E-05
Firmicutes/Clostridia/Clostridiales/Ruminococcaceae/Intestinimonas	0.0009	0.0021	0.0026	*ns*	*ns*	0.0004	0.0017
Firmicutes/Clostridia/Clostridiales/Lachnospiraceae/Acetatifactor/Species 1	*ns*	*ns*	*ns*	*ns*	*ns*	*ns*	0.0012

Individual OTUs in which fenugreek administration reversed high fat diet-induced decreases in representation were correlated against measures of hyperlipidemia. Data are p values of Pearson correlation with total cholesterol (mg/dl), low-density lipoprotein (LDL Chol.; mg/dl), high-density lipoprotein (HDL Chol.; %TC), body weight (grams), body fat (grams), fasting blood glucose (mg/dl), and glucose tolerance (blood glucose levels 40 minutes after oral loading). See Supplementary Table 1 for additional details (log2FC and Pearson r values) on HFD-decreased, fenugreek corrected taxa.

**Table 4:** OTUs that predicts metabolic decline in high fat-fed mice.

Correlation (Pearson) of HFD-Increased, Fenugreek-Corrected OTU’s to Metrics of Metabolic Function							
Individual OTUsDecreased by High Fat Diet (HF) Corrected by FG	Total Chol.	LDL Chol.	HDL Chol.	Body Weight	Body Fat	Fasting Glucose	Glucose Tolerance
Firmicutes/Clostridia/Clostridiales/Lachnospiraceae/Clostridium_XlVa/Species 4	*ns*	4.9E-05	*ns*	*ns*	*ns*	*ns*	*ns*
Firmicutes/ClostridiaClostridiales/Ruminococcaceae/Anaerotruncus	0.0004	1.3E-08	*ns*	*ns*	0.0016	*ns*	4.6E-06
Firmicutes/Clostridia/Clostridiales/Lachnospiraceae/Clostridium_XlVa/Species 5	*ns*	5.8E-05	0.0024	*ns*	*ns*	*ns*	*ns*
Firmicutes/Clostridia/Clostridiales/Lachnospiraceae/Clostridium_XlVa/Species 6	0.0002	4.9E-09	0.0051	*ns*	*ns*	*ns*	0.0002
Bacteroidetes/Bacteroidia/Bacteroidales/Porphyromonadaceae/Barnesiella/Species 1	9.1E-05	4.1E-10	*ns*	0.0009	0.0009	0.0024	1.6E-06
Bacteroidetes/Bacteroidia/Bacteroidales/Porphyromonadaceae/Barnesiella/Species 2	0.0005	4.2E-09	*ns*	*ns*	*ns*	*ns*	8.8E-06
Firmicutes/Clostridia/Clostridiales/Lachnospiraceae/Clostridium_XlVa/Species 7	8.1E-07	5.7E-10	4.8E-05	*ns*	*ns*	*ns*	0.0001
Firmicutes/Clostridia/Clostridiales/Lachnospiraceae/Clostridium_XlVa/Species 8	0.0001	0.0002	0.0030	*ns*	1.2E-05	0.0011	1.3E-05
Firmicutes/Clostridia/Clostridiales/Lachnospiraceae/Clostridium_XlVa/Species 9	4.1E-06	5.9E-08	0.0007	*ns*	1.2E-06	4.1E05	*ns*
Firmicutes/Bacilli/Lactobacillales/Streptococcaceae/Streptococcus	*ns*	*ns*	*ns*	*ns*	*ns*	*ns*	0.0007
Firmicutes/Clostridia/Clostridiales/Lachnospiraceae/Clostridium_XlVa/Species 10	0.0009	2.1E-05	*ns*	*ns*	0.0002	*ns*	5.0E-06
Firmicutes/Bacilli/Lactobacillales/Lactobacillaceae/Lactobacillus/Species 1	4.2E-06	*ns*	*ns*	7.1E-08	4.3E-09	5.6E07	1.1E-06
Firmicutes/Bacilli/Lactobacillales/Lactobacillaceae/Lactobacillus/Species 2	1.3E-06	0.0001	*ns*	2.9E-08	2.1E-09	2.9E-07	5.2E-07
Actinobacteria/Actinobacteria/Coriobacteridae/Coriobacteriales/Coriobacterineae	*ns*	*ns*	*ns*	*ns*	*ns*	*ns*	0.0033
Firmicutes/Clostridia/Clostridiales/Lachnospiraceae/Roseburia	6.0E-05	5.4E-07	0.0004	*ns*	*ns*	*ns*	*ns*
Firmicutes/Clostridia/Clostridiales/Lachnospiraceae/Clostridium_XlVa/Species 11	*ns*	*ns*	*ns*	0.0030	0.0016	*ns*	0.0015
Firmicutes/Clostridia/Clostridiales/Clostridiales_Incertae_Sedis_XI/Dethiosulfatibacter		0.0039	*ns*	*ns*	0.0004	*ns*	*ns*
Bacteroidetes/Bacteroidia/Bacteroidales/Porphyromonadaceae/Barnesiella/Species 3	*ns*	1.4E-05	*ns*	*ns*	*ns*	*ns*	0.0040
Firmicutes/Clostridia/Clostridiales/Lachnospiraceae/Acetatifactor/Species 2	0.0003	0.0002	*ns*	*ns*	1.6E-05	*ns*	*ns*
Firmicutes/Clostridia/Clostridiales/Lachnospiraceae/Clostridium_XlVa/Species 12	5.2E-05	2.2E-07	0.0003	*ns*	*ns*	*ns*	*ns*

Individual OTUs in which fenugreek administration reversed high fat diet-induced increases in representation were correlated against measures of hyperlipidemia. Data are correlation coefficients (Pearson *r*) and p values of correlation with total cholesterol (mg/dl), low-density lipoprotein (LDL Chol.; mg/dl), high-density lipoprotein (HDL Chol.; %TC), body weight (grams), body fat (grams), fasting blood glucose (mg/dl), and glucose tolerance (blood glucose levels 40 minutes after oral loading). See Supplementary Table 2 for additional details (log2FC and Pearson r values) on HFD-increased, fenugreek corrected taxa.

## Discussion

While beneficial effects of fenugreek on hyperlipidemia and hyperglycemia have been widely reported, the mechanisms of fenugreek-mediated actions on metabolic function are unknown. Here we demonstrate the robust effects of fenugreek on gut microbiota, and describe a novel combination of statistical analyses including Unifrac, iterative DESeq. 2, and pairwise correlation matrices to generate insight into the role of intestinal microbial changes in the protective effects of fenugreek. Specifically, sequencing analyses reveal that fenugreek significantly increased overall microbiome diversity in mice, and specifically reversed the actions of high dietary fat on key intestinal taxa. Furthermore, the representation of fenugreek-corrected taxa significantly correlated with metabolic function, including changes in body weight and composition, glucose regulation, and hyperlipidemia.These findings are in agreement with the extensive body of literature documenting the ability of high fat diet to reduce bacterial diversity and disrupt the balance of pathogenic/commensal bacteria within the intestine^36,42,38,43^. As data from our lab and others show that this pattern of gut dysbiosis is sufficient to impair both metabolic and neurologic function^37,44–48^, data in this paper suggest that the reported effects of fenugreek on gut microbiota^33,34,40,49^, may be fundamental to its beneficial properties. In light of the stubborn prevalence of obesity and the pervasive accessibility of unhealthy diets, it is both clinically significant and generally promising that these data suggest that partial reversal of gut dysbiosis and metabolic impairment can be achieved with botanical supplementation within the context of unhealthy, Western-style diets.

While the association of intestinal dysbiosis with metabolic disease is well established^45–48^ causal relationships have not been identified and it is not understood how microbiome constituents impact metabolic resilience/vulnerability. Three major phyla are the most abundant in the human distal intestine: Bacteroidetes (gram-negative), Firmicutes (gram-positive), and Actinobacteria (gram-positive). Early studies suggested that obesity causes reductions in Bacteroidetes and increases in Firmicutes^38,43^ and indeed further studies indicate that these changes are reversed with weight loss^50,51^. While these data suggest that the balance between these phyla might broadly impact host physiology, this binary distinction does not always occur^52,53^ and may be too simple to faithfully reflect the complexity of diet-induced changes to the gut microbiome^54–56^. In the present study as well as our previous studies^54,57^, differences between control and high fat groups did not manifest as phylum-level shifts but rather differential representation *within* taxa, particularly Firmicutes. Indeed, the majority of taxa included in Tables 3 and 4 arise from the Clostridium class (*Clostridium cluster XIVa*) or the *Clostridium leptum* group (*Clostridium cluster IV*). This is notable, as divergent shifts in the representation of Clostridia have been reported in other pathophysiological conditions. For example, while overall Clostridium representation generally increases with age, *Clostridium XIVa* clusters have been shown to be significantly reduced in the elderly^58^. The bacteria in *Clostridium XIVa* play major roles in the fermentation of carbohydrates within the gut^59^, and the major end products of hind-gut fermentation are short-chain fatty acids (SCFAs). Further, Fermicutes produce primarily butyrate as their metabolic end product^60^, and butyrate is the main source of nutrition for gut epithelium cells^61^. Depletion of butyrate is associated with impaired intestinal barrier integrity^62^, and loss of intestinal barrier function is in turn associated with a growing number of inflammatory disease states diseases, including obesity as well as autoimmune diseases and cancer (reviewed in^63,64^). While butyrate was not directly measured in the present study, decreased levels of butyrate and other SCFA are widely reported in the context of obesity while high-fiber plant products are known to increase colonic fermentation and the generation of SCFA^65^.

Our data indicate that fenugreek is particularly effective against hyperlipidemia, which is in keeping with results of both experimental and clinical studies^14,22,30,66^. While there are several mechanisms whereby changes in gut microbiota could mediate the effects of fenugreek on serum lipids, most ultimately impact the absorption of dietary fat. For example, intestinal microbiota alter the metabolism of diet-derived long-chain fatty acids such as conjugated linoleic acid, modulating absorption^67^. Gut microbiota can also moderate cholesterolemia by regulating cholesterol conversion into coprostanol^68^. Dietary cholesterol is largely incorporated into chylomicrons for absorption in the small intestine. However, significant quantities of cholesterol (as much as 1 gram per day) escape proximal absorption to enter the colon to be either be excreted or absorbed. Microbial-based metabolism of cholesterol into coprostanone/coprostanol reduces blood cholesterol by increasing fecal cholesterol excretion^69,70^. Interestingly, recent data suggest that taxa arising from Lachnospiraceae and Runinococcacea families of the phylum Fermicutes are uniquely associated with high coprostanol generation in healthy humans^71^, while other studies likewise link these gut microbiota to variation in blood lipid levels independently of age, sex, and host genetics^72^. It is important to note that seven of the eight of the taxa listed in Table 3 are members of Lachnospiraceae and Runinococcacea families, suggesting that reversal of HFD-induced decreases in these key taxa by fenugreek could remediate hyperlipidemia by promoting fecal fat excretion. In further support of this scenario, published data suggest that fenugreek supplementation can dose-dependently increase fecal excretion of cholesterol from rats given high fat/high calorie diets^73^. Collectively, these data raise the possibility that increased representation of coprostanoligenic taxa arising from Lachnospiraceae and Runinococcacea families could participate in the lipid-lowering effects of fenugreek, and further suggest that identification and analysis such strains could lead to improved understanding and management of hypocholesteremia.

Transformation and metabolism of bile acids is another key pathway whereby fenugreek-shaped intestinal bacteria could impact serum lipids (reviewed in^74^). Indeed, fenugreek has been reported to inhibit the intestinal absorption of primary and secondary bile acids^75^, and to increase bile acid excretion into feces^30^. While the bulk of bile acids released into the intestine are efficiently absorbed and recycled back to the liver, perhaps 5% of the total bile acid pool progresses into the colon. Bile acids reaching the colon are subject to several microbial-mediated reactions including transformation into secondary bile acids by dihydroxylation, and deconjugation by bile salt hydrolases. While data indicate that bile salt hydrolases are a pervasive microbial adaptation to the human gut environment with enrichment in major genera including *Bacteroides, Clostridium, Lactobacillus*, and *Eubacterium*^76^, probiotics with bile salt hydrolytic activity can lower serum cholesterol^77,78^. With regard to more broad metabolic benefits, secondary bile acids generated by microbial metabolism are potent ligands of the G-protein coupled receptor TGR5, the activation of which triggers release of GLP-1 and insulin, thereby modulating host glucose tolerance and energy expenditure^79^.

While this study is in keeping with an extensive body of literature on the beneficial effects of fenugreek, the use of whole seed supplementation precludes identification of the bioactive constituent(s) mediating changes to gut microbiota. Furthermore, only male mice were used, so sex-based differences in the effects of fenugreek or the relation of such to metabolic function cannot be resolved. This point is especially significant as female C57BL/6J mice are generally considered resistant to diet induced obesity^80,81^. Notwithstanding these limitations, these data indicate that fenugreek supplementation can stabilize metabolic function within the context of high fat consumption. Indeed, fenugreek did not affect food intake or alleviate diet-induced obesity, but rather was able to bolster resistance of the obese mice to hyperlipidemia and glucose intolerance. We use the term “metabolic resiliency” to describe this ability to preserve, at least in part, a healthy metabolic phenotype in the context of powerful external stressors – in this case sustained consumption of a high fat diet and the obese state. This is a significant finding, as while the components of a healthy lifestyle are generally well known, numerous societal factors including poverty, food deserts, irregular/sedentary work schedules combine to hinder a consistently healthy lifestyle for most Americans. Thus, use of fenugreek and/or other strategies to maintain a healthy population of intestinal microbes in the context of a high fat diet could foster metabolic resilience even when diets/lifestyles are not optimal. Indeed, while fenugreek conferred protection against hyperlipidemia and glucose intolerance, it is important to note that representation of specific fenugreek-corrected taxa correlated frequently with aspects of metabolic function (e.g., body weight, body fat, total cholesterol, fasting blood glucose) that were not significantly improved in fenugreek-treated mice (see Tables 3 and 4). While correlation does not equal causation, these data clearly illustrate the very close relationship of individual microbes and microbial balance with metabolic function, and raise the possibility that strategic manipulation of key intestinal taxa could result in a more complete reversal of the adverse action of high fat diet. Thus, the action of fenugreek could be possibly optimized by dose, preparation, or combination with additional factors (probiotics, etc) to have a greater effect on key microbiota, enhancing its beneficial profile. These data also suggest that perhaps intestinal microbial “fingerprints” could be generated to estimate vulnerability to metabolic dysfunction and/or the potential for efficacy of metabolic interventions. Overall, data in the manuscript strongly suggest that the development of microbially-targeted therapies – both primary and adjunctive – that are built upon safe, natural, plant-based products like fenugreek could be used to attain significant advancements in public health within the context of contemporary dietary environments.

## Materials and Methods

### Animals and diets

This study was carried out in strict accordance with PHS/NIH guidelines on the use of experimental animals, and all experimental protocols were approved by the Institutional Animal Care and Use Committee at Pennington Biomedical Research Center. Data in this manuscript follows an initial publication on the effects of whole fenugreek seed supplementation (2% w/w) on overall metabolic function in the context of a 16-week trial of high fat diet consumption^41^. As detailed in our initial report^41^, male, 9 week-old C57BL/6 J mice were purchased from Jackson Laboratories, and group-housed (4/cage) under standard conditions with *ad libitum* access to food/water. After 7 days acclimation, mice were randomly separated into the following 4 groups (20 mice each in each group): high fat diet ± fenugreek (HFD and HFD/FG) and control diet ± fenugreek (CD and CD/FG) for 16 weeks. HFD and HFD/FG mice were fed a diet with 60% kcal from fat without or with 2% fenugreek seed powder incorporated into the diets, respectively (Research Diets Inc. D12492, D16020410), while CD mice were fed a nutritionally matched control low fat diet (10% kcal from fat) with or without 2% fenugreek seed powder (Research Diet Inc.; D12450J, D16020408). All diets contained 10% kcal from protein with the balance in caloric intake provided by differences in carbohydrate content. *T. foenum-graecum L*. “Fenugreek” seeds were purchased from Johnny’s Selected Seeds, Winslow Maine, certified organic for sprouting. Fenugreek seeds were ground in the Department of Plant Biology at Rutgers University and hand-delivered to Research Diets Inc., (New Brunswick, NJ) for commercial incorporation into treatment diets at 2% of the diet by weight.

### Metabolic phenotyping

Assessment of metabolic function is fully detailed in our previous report^41^. Briefly, body composition (fat mass, fat-free/lean mass, and water content) was measured by briefly placing in mice into Bruker minispec LF110 time domain NMR analyzer (Bruker Optics, Billerica MA) as described previously^41^. Glucose tolerance was measured using an oral glucose tolerance assay (OGTT) based on repeated sampling of tail blood using a glucometer (Ascensia Elite, Bayer, Mishawaka, IN) at 0, 20, 40, 60, and 120 minutes after oral glucose (2 gm/kg) administration. All mice remained in the study for the duration of the 16-week feeding trail, after which mice with euthanized following a 8-hr fast by decapitation under deep isoflurane anesthesia. Levels of total cholesterol, HDL cholesterol, LDL cholesterol, and triglycerides in serum collected at euthanasia were measured colorimetrically (Wako Chemicals, Richmond, VA).

### 16S Metagenomic sequencing

Fecal samples were collected at euthanasia, and DNA preparation, sequencing and bioinformatics were performed by the PBRC Genomics Core Facility. DNA was isolated using a commercial reagent system (MoBio Power Fecal Kit, MoBio Laboratories, Carlsbad, CA) augmented by enzymatic lysis using lysostaphin, mutanolysin, and lysozyme^82^. Sequencing libraries targeting V4 of the gene encoding the 16S ribosomal RNA were generated using a commercially available kit (NEXTflex™ 16S V4 Amplicon-Seq Library Prep Kit, BIOO Scientific, Austin, TX), relying on 16S gene-specific primer sequences V4F 5′-GTGCCAGCMGCCGCGGTAA-3′ and V4R 5′-GGACTACHVGGGTWTCTAAT-3′, and including Illumina adaptors and molecular barcodes as described by the manufacturer to produce 253 bp amplicons. Samples were sequenced with custom primers (BIOO Scientific, Austin, TX) on an Illumina MiSeq instrument using version 3 sequencing chemistry (300 bp paired end reads). Forward and reverse sequence reads were processed into double-stranded DNA contigs using quality control metrics implemented in the software package ‘mothur’^83^. Sequence clustering (at better than 97% identity) to identify operational taxonomical units (OTUs), removal of chimeric sequences, and generation of a read count table (i.e. tabulating the occurrence of each OTU in each sample) were performed with the software package ‘usearch’^84^. Taxonomical classification of each OTU sequence relied on the SILVA 16S rRNA sequence database version 123.1^85^, and statistical tests for differential representation were performed with tools incorporated in ‘mothur’, as well as using the software package DESeq. 2^86^. Relative abundance of each OTU was examined on the phylum, class, order, family and genus levels.

### Statistical analyses

Biochemical data were analyzed using Prism software (GraphPad Software, Inc.), and displayed as mean ± standard error, and were analyzed by ANOVA. Statistical significance for all analyses was accepted at p < 0.05, and *, **, and *** represent p < 0.05, p < 0.01, and p < 0.001, respectively. Alpha diversity (chao1 metrics) and beta diversity (weighted UniFrac metrics^87^) were assessed using tools implemented in ‘mothur’ on the basis of 80,000 sequences per sample. Differential representation of OTUs was assessed using DESeq. 2 on the basis of sequence count data, relying on Wald statistics with Benjamini-Hochberg correction and a false discovery rate cutoff set at 0.1. Inter-sample relationships relying on Principal Component Analysis on the basis of DESeq. 2 output, and data visualizations were both performed using JMP Genomics software (SAS, Cary, NC). Pairwise Pearson correlations of individual metrics of metabolic function against individual HFD-transformed, fenugreek-corrected OTU expression were carried out using Prism software.

## Supplementary information

Supplementary File.
