# Supplementary material for: Fenugreek Counters the Effects of High Fat Diet on Gut Microbiota in Mice: Links to Metabolic Benefit
Source: Sci Rep. 2020 Jan 27;10:1245. doi: 10.1038/s41598-020-58005-7 (PMC6985225; doi:10.1038/s41598-020-58005-7)
Supplement: Supplementary file 1 — Supplementary File. [file 41598_2020_58005_MOESM1_ESM.docx]

**Supplementary Datae for SREP-19-27170 "Fenugreek Counters the Effects of High Fat Diet on Gut Microbiota in Mice: Links to Metabolic Benefit”**

Supplementary Tables that include Log2(fold changes) and Pearson r values for HFD-decreased, fenugreek corrected taxa (Supplementary Table 1) and HFD-increased, fenugreek corrected taxa (Sepplementary Table 2).

|  | | | | | | | **Relation to Metabolic Phenotype (p values/ Pearson *r*)** | | | | | | | | | | | | | | |  |  |
| --- | --- | --- | --- | --- | --- | --- | --- | --- | --- | --- | --- | --- | --- | --- | --- | --- | --- | --- | --- | --- | --- | --- | --- |
| **OTUs**  **Decreased by High Fat Diet (HF)** | | | **Log2FC LF to**  **HF** | | **Log2FC HF to HFFG** | | **TC** | | **LDL** | | **HDL** | | **BW** | | **BFat** | | **FBG** | | **GT** | | |  |  |
| Firmicutes/Clostridia/Clostridiales/  Lachnospiraceae/Clostridium_XlVa/  Species 1 | | | -4.224 | | 3.466 | | *ns* | | *ns* | | *ns* | | *ns* | | 0.0014  -0.3899 | | *ns* | | 0.0001  -0.3899 | | |  |  |
| Firmicutes/Clostridia/Clostridiales/  Lachnospiraceae/Clostridium_XlVa/  Species 2 | | | -3.708 | | 1.622 | | *ns* | | *ns* | | *ns* | | 0.0027  -0.3694 | | 0.0025  -0.3712 | | *ns* | | *ns* | | |  |  |
| Firmicutes/Clostridia/Clostridiales/  Lachnospiraceae/Clostridium_XlVa/  Species 3 | | | -3.111 | | 1.561 | | 0.0001  -0.4497 | | 0.0034  -0.3668 | | 0.0011  0.4039 | | *ns* | | *ns* | | 0.0006  -0.4193 | | 0.0001  -0.4193 | | |  |  |
| Firmicutes/Clostridia/Clostridiales/  Ruminococcaceae/Flavonifractor | | | -2.980 | | 2.789 | | 1.2E-05  -0.5167 | | 0.0005  -0.4320 | | 0.0001  0.4636 | | 1.6E-06  -0.5590 | | 2.1E-06  -0.5534 | | 2.2E-05  -0.5038 | | 1.9E-05  -0.5063 | | |  |  |
| Firmicutes/Erysipelotrichia/Erysipelotrichales/Erysipelotrichaceae/  Turicibacter | | | -2.892 | | 2.722 | | 0.0004  -0.4302 | | *ns* | | 0.0024  0.3787 | | *ns* | | *ns* | | *ns* | | 0.0015  -0.3898 | | |  |  |
| Firmicutes/Clostridia/Clostridiales/  Ruminococcaceae/Oscillibacter | | | -2.447 | | 1.534 | | 0.0002  -0.4443 | | *ns* | | 1.3E-05  0.5223 | | *ns* | | 4.0E-05  -0.4896 | | 0.0002  -0.4478 | | 4.2E-05  -0.4887 | | |  |  |
| Firmicutes/Clostridia/Clostridiales/  Ruminococcaceae/Intestinimonas | | | -1.837 | | 2.556 | | 0.0009  -0.4020 | | 0.0021  -0.3839 | | 0.0026  0.3765 | | *ns* | | *ns* | | 0.0004  -0.4308 | | 0.0017  -0.3851 | | |  |  |
| Firmicutes/Clostridia/Clostridiales/  Lachnospiraceae/Acetatifactor/  Species 1 | | | -1.782 | | 3.022 | | *ns* | | *ns* | | *ns* | | *ns* | | *ns* | | *ns* | | 0.0012  -0.3965 | | |  |  |
|  | | | | |  |  |  |  |  |  |  |  |  |  |  |  |  |  |  |  |  |  |  |
|  | | | | | | | | | | | | | | | | | | | |  |  |  |  |
| **Supplementary Table 1. OTUs that predicts metabolic decline in high fat-fed mice.** Individual OTUs in which fenugreek administration reversed high fat diet-induced decreases in representation were correlated against measures of hyperlipidemia. Log2-transformed fold-changes (Log2FC) in representation are shown for comparison of LF to HF and for HF to HFFG. Data are correlation coefficients (Pearson *r*) and p values of correlation with total cholesterol (TC; mg/dl), low-density lipoprotein (LDL; mg/dl), high-density lipoprotein (HDL; %TC), body weight (BW; grams), body fat (BFat; grams), fasting blood glucose (FBG; mg/dl), and glucose tolerance (GT; blood glucose levels 40 minutes after oral loading). | | | | | | | | | | | | | | | | | | | |  |  |  |  |
|  | | | | | | | | | **Relation to Metabolic Phenotype (p values/ Pearson R)** | | | | | | | | | | | | | | |
| **OTUs**  **Increased by High Fat Diet (HF)** | | | | | **Log2FC LF to**  **HF** | | **Log2FC HF to HFFG** | | **TC** | | **LDL** | | **HDL** | | **BW** | | **BFat** | | **FBG** | | **GT** | | |
| Firmicutes/Clostridia/Clostridiales/  Lachnospiraceae/Clostridium_XlVa/  Species 4 | | | | | 4.881 | | -2.214 | | *ns* | | 4.9E-05  0.4920 | | *ns* | | *ns* | | *ns* | | *ns* | | *ns* | | |
| Firmicutes/ClostridiaClostridiales/Ruminococcaceae/Anaerotruncus | | | | | 4.330 | | -2.247 | | 0.0004  0.4330 | | 1.3E-08  0.6476 | | *ns* | | *ns* | | 0.0016  0.3865 | | *ns* | | 4.6E-06  0.5376 | | |
| Firmicutes/Clostridia/Clostridiales/Lachnospiraceae/Clostridium_XlVa/Species 5 | | | | | 4.149 | | -2.494 | | *ns* | | 5.8E-05  0.4879 | | 0.0024  -0.3792 | | *ns* | | *ns* | | *ns* | | *ns* | | |
| Firmicutes/Clostridia/Clostridiales/Lachnospiraceae/Clostridium_XlVa/Species 6 | | | | | 4.025 | | -3.271 | | 0.0002  0.4470 | | 4.9E-09  0.6614 | | 0.0051  -0.3518 | | *ns* | | *ns* | | *ns* | | 0.0002  0.4534 | | |
| Bacteroidetes/Bacteroidia/Bacteroidales/  Porphyromonadaceae/Barnesiella  /Species 1 | | | | | 3.792 | | -2.053 | | 9.1E-05  0.4694 | | 4.1E-10  0.6936 | | *ns* | | 0.0009  0.4042 | | 0.0009  0.4044 | | 0.0024  0.3736 | | 1.6E-06  0.5593 | | |
| Bacteroidetes/Bacteroidia/Bacteroidales/  Porphyromonadaceae/Barnesiella  /Species 2 | | | | | 3.648 | | -3.597 | | 0.0005  0.4207 | | 4.2E-09  0.6633 | | *ns* | | *ns* | | *ns* | | *ns* | | 8.8E-06  0.5241 | | |
| Firmicutes/Clostridia/Clostridiales/Lachnospiraceae/Clostridium_XlVa/Species 7 | | | | | 3.374 | | -2.038 | | 8.1E-07  0.5716 | | 5.7E-10  0.6897 | | 4.8E-05  -0.4922 | | *ns* | | *ns* | | *ns* | | 0.0001  0.4654 | | |
| Firmicutes/Clostridia/Clostridiales/Lachnospiraceae/Clostridium_XlVa/Species 8 | | | | | 2.828 | | -2.345 | | 0.0001  0.4627 | | 0.0002  0.4616 | | 0.0030  -0.3707 | | *ns* | | 1.2E-05  0.5173 | | 0.0011  0.3986 | | 1.3E-05  0.5151 | | |
| Firmicutes/Clostridia/Clostridiales/Lachnospiraceae/Clostridium_XlVa/Species 9 | | | | | 2.795 | | -1.646 | | 4.1E-06  0.5401 | | 5.9E-08  0.6242 | | 0.0007  -0.4204 | | *ns* | | 1.2E-06  0.5642 | | 4.1E05  0.4894 | | *ns* | | |
| Firmicutes/Bacilli/Lactobacillales/Streptococcaceae/Streptococcus | | | | | 2.686 | | -4.685 | | *ns* | | *ns* | | *ns* | | *ns* | | *ns* | | *ns* | | 0.0007  0.4133 | | |
| Firmicutes/Clostridia/Clostridiales/Lachnospiraceae/Clostridium_XlVa/Species 10 | | | | | 2.657 | | -3.090 | | 0.0009  0.4047 | | 2.1E-05  0.5126 | | *ns* | | *ns* | | 0.0002  0.4432 | | *ns* | | 5.0E-06  0.5361 | | |
| Firmicutes/Bacilli/Lactobacillales/Lactobacillaceae/Lactobacillus/Species 1 | | | | | 2.512 | | -1.007 | | 4.2E-06  0.5395 | | *ns* | | *ns* | | 7.1E-08  0.6134 | | 4.3E-09  0.6550 | | 5.6E07  0.5784 | | 1.1E-06  0.5654 | | |
| Firmicutes/Bacilli/Lactobacillales/Lactobacillaceae/Lactobacillus/Species 2 | | | | | 2.370 | | -1.096 | | 1.3E-06  0.5630 | | 0.0001  0.4703 | | *ns* | | 2.9E-08  0.6273 | | 2.1E-09  0.6650 | | 2.9E-07  0.5898 | | 5.2E-07  0.5798 | | |
| Actinobacteria/Actinobacteria/Coriobacteridae/Coriobacteriales/Coriobacterineae | | | | | 1.905 | | -2.834 | | *ns* | | *ns* | | *ns* | | *ns* | | *ns* | | *ns* | | 0.0033  0.3622 | | |
| Firmicutes/Clostridia/Clostridiales/Lachnospiraceae/Roseburia | | | | | 1.847 | | -1.591 | | 6.0E-05  0.4800 | | 5.4E-07  0.5867 | | 0.0004  -0.4392 | | *ns* | | *ns* | | *ns* | | *ns* | | |
| Firmicutes/Clostridia/Clostridiales/Lachnospiraceae/Clostridium_XlVa/Species 11 | | | | | 1.843 | | -1.117 | | *ns* | | *ns* | | *ns* | | 0.0030  0.3658 | | 0.0016  0.3862 | | *ns* | | 0.0015  0.3897 | | |
| Firmicutes/Clostridia/Clostridiales/Clostridiales_Incertae_Sedis_XI/  Dethiosulfatibacter | | | | | 1.469 | | -0.678 | | *ns* | | 0.0039  0.3614 | | *ns* | | *ns* | | 0.0004  0.4285 | | *ns* | | *ns* | | |
| Bacteroidetes/Bacteroidia/Bacteroidales/  Porphyromonadaceae/Barnesiella  /Species 3 | | | | | 1.351 | | -2.335 | | *ns* | | 1.4E-05  0.5216 | | *ns* | | *ns* | | *ns* | | *ns* | | 0.0040  0.3550 | | |
| Firmicutes/Clostridia/Clostridiales/Lachnospiraceae/Acetatifactor/Species 2 | | | | | 1.204 | | -0.925 | | 0.0003  0.4353 | | 0.0002  0.4620 | | *ns* | | *ns* | | 1.6E-05  0.5115 | | *ns* | | *ns* | | |
| Firmicutes/Clostridia/Clostridiales/Lachnospiraceae/Clostridium_XlVa/Species 12 | | | | | 1.192 | | -0.601 | | 5.2E-05  0.4833 | | 2.2E-07  0.6020 | | 0.0003  -0.4442 | | *ns* | | *ns* | | *ns* | | *ns* | | |

**Supplementary Table 2. OTUs that predicts metabolic decline in high fat-fed mice.** Individual OTUs in which fenugreek administration reversed high fat diet-induced increases in representation were correlated against measures of hyperlipidemia. Log2-transformed fold-changes (Log2FC) in representation are shown for comparison of LF to HF and for HF to HFFG. Data are correlation coefficients (Pearson *r*) and p values of correlation with total cholesterol (TC; mg/dl), low-density lipoprotein (LDL; mg/dl), high-density lipoprotein (HDL; %TC), body weight (BW; grams), body fat (BFat; grams), fasting blood glucose (FBG; mg/dl), and glucose tolerance (GT; blood glucose levels 40 minutes after oral loading).
